# Early-Phase Intervention With Traditional Japanese Acupuncture and Moxibustion for Fibromyalgia: A Case Report

**DOI:** 10.7759/cureus.74385

**Published:** 2024-11-25

**Authors:** Takuya Masuda, Kenichiro Egawa, Yu Takeshita, Koichiro Tanaka

**Affiliations:** 1 Division of General Internal Medicine & Rheumatology, Mitsui Memorial Hospital, Tokyo, JPN; 2 Department of Traditional Medicine, Toho University, Tokyo, JPN; 3 Department of Western Medicine, Hokushin-kai, Academic Society of Traditional Japanese Acupuncture and Moxibustion, Osaka, JPN; 4 Division of Palliative Care, Mitsui Memorial Hospital, Tokyo, JPN; 5 Department of Integrative/Complementary Medicine, Acupuncture Clinic, Seimei-in, Tokyo, JPN

**Keywords:** early-phase intervention, fibromyalgia, hokushin-kai style, selective serotonin reuptake inhibitor (ssri), traditional japanese acupuncture and moxibustion

## Abstract

Fibromyalgia (FM) is a common chronic pain with no established treatment. Acupuncture is an expected treatment for FM though a diagnosis of FM tends to be delayed, and the advantage is still unclear in early-phase intervention with acupuncture treatment for FM.

A 51-year-old woman with panic disorder presented with a four-month history of whole-body pain and was diagnosed with FM. She was unable to do housework or control her pain with medication. And she took 10 mg of paroxetine, a selective serotonin reuptake inhibitor (SSRI), to treat her depressive mood or panic disorder. A traditional Japanese acupuncture and moxibustion treatment, *Hokushin-kai*, was started. According to the Oriental medical diagnosis, she was categorized with “hyperactivity of liver yang,” “dampness encumbering spleen,” and “kidney yin deficiency” patterns. The treatment was administered once a week, and only one or two sterilized disposable needles were inserted into each acupoint (such as BL19, LR8, or KI3) for 10 minutes and with no manipulations. At the first presentation, the numerical rating scale (NRS) value was 6. After six weeks, her whole-body pain level remained around NRS 0-2 for 4-5 days after each treatment session. She was then able to resume housework after 12 weeks.

Since ancient times, acupuncturists have recognized how acupuncture becomes less effective over time, especially for chronic pain. Moreover, the concept of a treatment-sensitive period for chronic pain prevention has recently been proposed. Further research, including early-phase interventions for acupuncture treatment, is required to evaluate the clinical effects of various treatments on FM.

## Introduction

There is no established treatment for fibromyalgia (FM), a common form of chronic pain with an estimated prevalence of 2-3% worldwide [1]. Recently, clinical studies of acupuncture were shown to improve pain and stiffness in patients with FM, and the European League Against Rheumatism (EULAR) recommended acupuncture as one of the first-line non-pharmacological treatments for FM in 2017 [2]. Almost all previous studies of acupuncture for FM citing this recommendation reviewed Western or traditional Chinese medicine (TCM) methods of acupuncture.

Meanwhile, a diagnosis of FM tends to be delayed [3]. In a Japan Fibromyalgia Support Association (JFSA) questionnaire survey [4] (n=339), 11.5% of respondents stated that an FM diagnosis was made within a period of several months: 14.4% within one year, 18.9% within 1-2 years, and 52.5% over two years. It is speculated that the reason for this delayed diagnosis is that some medical doctors find it hard to recognize that the pain is being caused by FM, or that such patients fall into a state of ‘doctor shopping’ while seeking a satisfactory diagnosis.

Since ancient times, many acupuncturists have recognized that acupuncture tends to become less effective over time, especially for chronic pain. Previous studies revealed that acupuncture has some clinical effects but does not relieve pain completely in FM patients with long-term (around 10 years) pain [5,6]. Moreover, there is no evidence demonstrating any advantage in early-phase intervention with acupuncture treatment for FM [1].

Acupuncture and moxibustion are freely accessible in Japan, and the JFSA questionnaire survey [4] (n=538) showed that 44.4% of respondents visited a hospital, 31% visited a clinic, and 8.6% visited an acupuncture clinic. Hence, it is speculated that some FM patients were treated with a traditional (inherited from ancient Japan) Japanese acupuncture method at a relatively early stage.

However, the traditional Japanese acupuncture technique is not widely known, and its clinical effects have not been evaluated by clinical trials. In this report, we describe a case of early-phase treatment of an FM patient using the *Hokushin-kai* style [7], one of the traditional Japanese acupuncture and moxibustion treatments.

## Case presentation

A 51-year-old woman presented with a four-month history of whole-body pain. One year earlier, she had visited our hospital for transient fever and cervical lymphadenopathy. At that time, laboratory data showed ANA 160× (homogeneous, speckled), positive for Anti-SS-A and Anti-ds DNA antibodies. However, as she did not meet the criteria of Sjögren’s syndrome or systemic lupus erythematosus (SLE), she was placed under observation. Four months before the presentation (in the summer), she experienced whole-body pain, depressive mood, and anxiety. She ceased work due to the pain. She complained about her pain during regular outpatient visits and palliative care at our hospital, where she sought pain management. She had no fever symptoms, dryness of the eyes and mouth, Raynaud’s phenomenon, or sensitivity to sunlight.

She had a medical history of panic disorder, ovarian cysts, endometriosis, and cerebral aneurysms. Administered medications included bisoprolol. There were no known drug allergies. The patient had worked part-time. She drank alcohol occasionally, had a seven-pack-year smoking history, and quit two years before this presentation. Her parents had cerebrovascular disease and no history of collagen disease.

**Figure 1:**
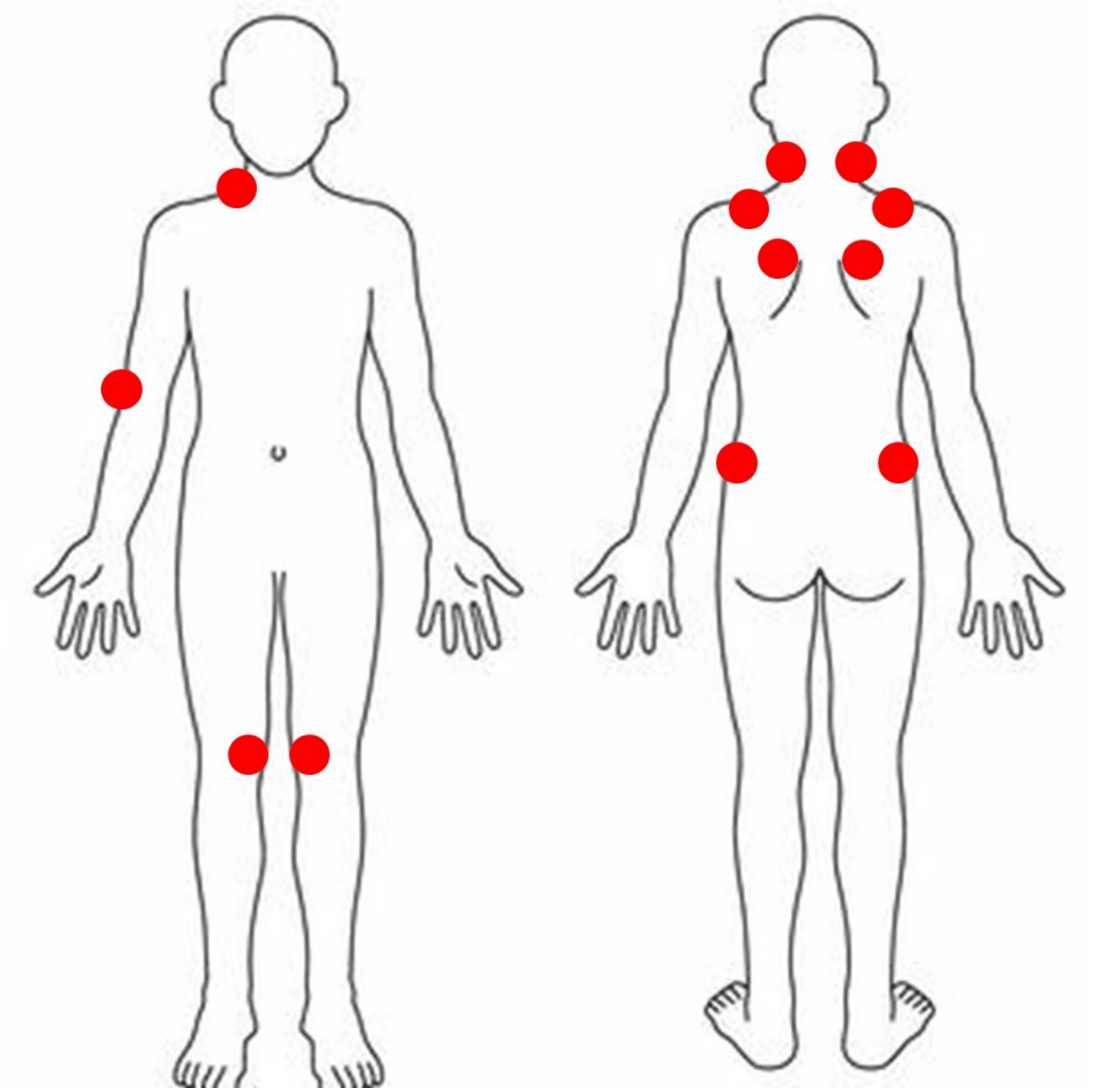
Tender points at the presentation There were multiple tender points on both sides of the back of the neck in the muscles at the base of the skull, trapezius muscles of the back shoulders, supraspinatus muscles in the shoulder blade area, upper-outer quadrant of the gluteal muscle of the buttocks, inner knee, and right side of the lower-front muscles of the neck, and the outside of the elbow. These multiple tender points are required to apply the fibromyalgia diagnostic criteria of the American College of Rheumatology 1990 [8]. The figure is the own creation of the authors

A physical examination revealed multiple tender points at the muscles at the base of the skull on both sides of the back of the neck, as well as the trapezius muscles of the back shoulders, supraspinatus muscles in the shoulder blade area, upper-outer quadrant of the gluteal muscle of the buttocks, inner knee, right side of lower-front muscles of the neck, and outside of the elbow (Figure 1). No joint tenderness or swelling, lymphadenopathy, oral ulcers, hair loss, or skin rash was seen.

Laboratory findings were as follows: WBC 3.7×10^3^/μL, Hb 11.8 g/dL, PLT 23×10^4^/μL, Cr 0.53 mg/dL, LD 132 U/L, CRP 0.00 mg/dL, C3 68 mg/dL, C4 7 mg/dL, ANA (homo, speckled) 160×, anti-ds-DNA antibody 14 IU/mL, and negative results for urine protein or casts. Other data are presented in Table 1, Table 2. Gadolinium scintigraphy showed no evidence of inflammation or bone lesions.

**Table 1:** Laboratory data (CBC, urinalysis, and coagulation system test) on the presentation of consultation for pain management CBC, complete blood count; ALP, alkaline phosphatase; APTT, activated partial thromboplastin time; MCV, mean corpuscular volume

CBC and coagulation system test			Reference range
WBC	3.7	×10^3^/μL	3.3-8.6
Neu	61.6	%	34-72
Lymph	34.4	%	18-48
Mono	5.8	%	4.3-10
Eosi	0.9	%	0.4-7.8
Baso	0.5	%	0.2-1.2
Hb	11.8	g/dL	13.7-16.8
MCV	95.9	fL	83-98
Plt	23.0	×10^4^/μL	16-34
PT-INR	0.92		0.85-1.15
APTT	32.6	sec	20-40
Urinalysis			
Gravity	1.004		
pH	6.0		4.6-8.0
Protein	(-)		
WBC	1-4	/HPF	<5
RBC	1-4	/HPF	<5
Cast	(-)		

**Table 2:** Laboratory data (blood chemistry and immunological tests plus) on the presentation of consultation for pain management The results were almost normal, except for the immune system. ESR, erythrocyte sedimentation rate; ANA, antinuclear antibody; RPR, rapid plasma reagin test; TP, treponema pallidum hemagglutination test.

Blood chemistry and immunological tests plus			Reference range
TP	7.1	g/dL	6.6-8.1
Alb	4.2	g/dL	4.1-5.1
GLU	89	mg/dL	73-109
BUN	10	mg/dL	8-20
Cre	0.53	mg/dL	0.65-1.07
LD	132	mg/dL	124-222
Na	142	mmol/L	138-145
K	4.4	mmol/L	3.6-4.8
Cl	110	mmol/L	101-108
Ca	9.2	mg/dL	8.8-10.1
T-bil	0.9	mg/dL	0.4-1.5
AST	14	U/L	13-30
ALP	53	U/L	10-42
CRP	0.00	mg/dL	0-0.14
ESR	6	mm/hr	0-10
TSH	1.949	µIU/mL	0.38-5.38
FT4	1.0	ng/dL	0.7-1.5
CH50	28.0	CH50/mL	25-48
C3	68	mg/dL	86-160
C4	7	mg/dL	17-45
IgG	1544	mg/dL	820-1740
IgA	179	mg/dL	90-400
IgM	37	mg/dL	31-200
ANA	160×		<40
Homo	160×		
Speckled	160×		
Anti-ds-DNA antibody	14	IU/mL	0-12
Anti-SS-A antibody	> 240	U/ml	<7.0
Anti-SS-B antibody	2.8	U/mL	<7.0
RPR	(-)		
TP	(-)		

The patient met the FM criteria of the American College of Rheumatology (ACR) 1990 [8] (Stage 4, The Japanese version of the FM Impact Questionnaire: JFIQ 66 pt: staging and JFIQ provided by the FM guideline from Japan in 2017 [9]). The criteria of ACR 1900 were chosen for comorbidities of panic disorder and suspected SLE; 2000 mg of acetaminophen, 20 mg of duloxetine, and 16 units of neurotropin were ineffective in relieving her pain, and acupuncture treatment was started. A psychiatrist replaced duloxetine with 10 mg of paroxetine, a selective serotonin reuptake inhibitor (SSRI), to treat her depressive mood or panic disorder.

Physical examination (in Oriental medicine) findings were as follows: Pulse diagnosis: replete and slippery; tongue diagnosis: purple, teeth-marked, white thick coating, and sublingual vein distention; complexion (facial color) exam: liver and spleen, dark; kidneys, reddish; lower energizer, loss of gloss, lips purple.

Acupoint exam findings were as follows: HT7, right deficiency (D)/left excess (E); SI3, right E/left D; LR3, right E/left D; KI6, right E/left D; BL16, right D/left E; BL18, right D/left E; BL20, right D/left E; BL23. right D/left D.

Abdominal exam (*Mubun*-style) findings were as follows: tension in the right spleen and liver and deficiency in the lower abdomen.

According to the Oriental medical diagnosis (termed “pattern identification”), she was categorized with “hyperactivity of liver yang,” “dampness encumbering spleen,” and a “kidney yin deficiency” pattern. We treated the patient with *Hokushin-kai*, a traditional Japanese style of acupuncture and moxibustion [7]. The treatment session occurred once a week. One or two sterilized disposable needles (Seirin Co. Shizuoka, Japan or I’SSIN Co., Hyogo, Japan) were inserted into each acupoint at a depth of 4-10 mm for 10 minutes, with no manipulations. Acupoints, needle sizes, and the needling method of each acupuncture treatment session are shown in Table 3.

**Table 3:** Acupoint, needle size, needling method, and retention time of each acupuncture treatment session The three sizes of the needles used were diameter 0.18 mm and length 10 mm, diameter 0.2 mm and length 20 mm, and diameter 0.2 mm and length 40 mm. R, right; L, left; D, draining; T, tonifying method.

Number of treatments	Acupoint 1	Needle size (mm)	Needling method	Acupoint 2	Needle size (mm)	Needling method	Retention time (min)
1	R, SI3	0.18 x 10	D	R, KI6	0.18 x 10	T	10
2	R, BL19	0.2 x 40	T	L, KI3	0.20 x 20	T	10
3	R, BL19	0.2 x 40	T	L, KI3	0.20 x 20	T	10
4	L, ST25	0.2 x 20	D	R, SP4	0.2 x 20	T	10
5	L, ST27	0.2 x 20	D	R, SP4	0.2 x 20	T	10
6	L, BL19	0.2 x 20	D	R, ST36	0.2 x 20	T	10
7	R, BL21	0.2 x 20	D	L, KI6	0.18 x 10	T	10
8	L, LR8	0.2 x 20	T	–	–	–	10
9	L, TE5	0.2 x 20	T	L, SP6	0.2 x 20	D&T	10
10	L, BL19	0.2 x 40	D	L, BL62	0.2 x 20	D	10
11	R, BL21	0.2 x 40	D	L, KI6	0.18 x 10	T	10
12	R, SI3	0.18 x 10	D	L, KI3	0.2 x 20	T	10

**Figure 2:**
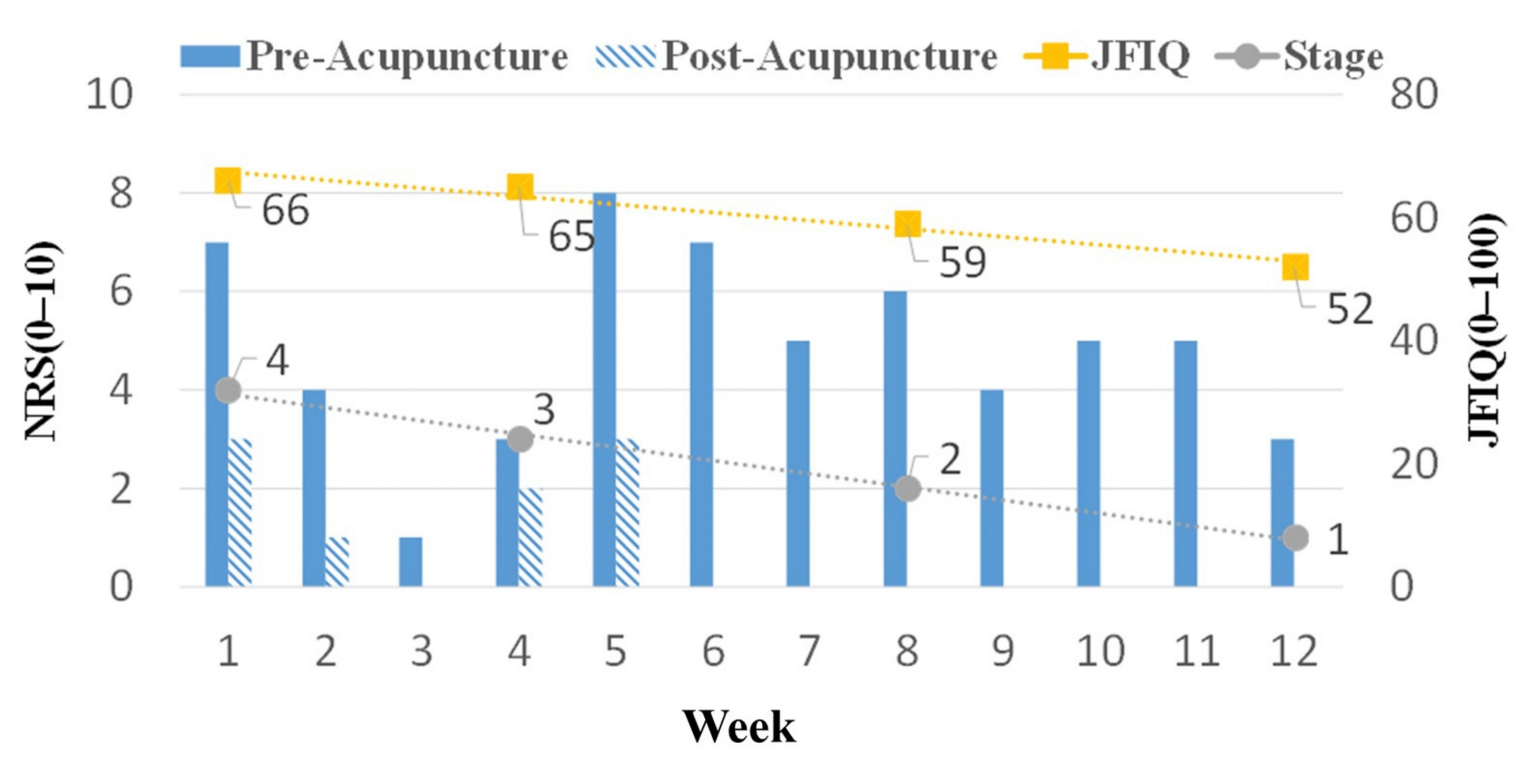
Therapeutic course of acupuncture treatment At first, the patient’s whole-body pain score was 6 on the numerical rating scale (NRS). There was one acupuncture treatment session per week. Whole-body pain improved to NRS 0–2 10 minutes after needle insertion (post-acupuncture). After six weeks, her whole-body pain level remained around NRS 0–2 for 4–5 days after each treatment session. Twelve weeks later, the NRS score of her whole-body pain and the staging improved to 3 and 1, respectively. She was able to resume housework. Although her depressive disorder and anxiety remained, JFIQ decreased to 52 pt.

At first, the numerical rating scale (NRS) score of her whole-body pain was 6. There was one acupuncture treatment session per week. Whole-body pain improved to NRS 0-2 10 minutes after needle insertion. She gradually became able to resume housework. After six weeks, her whole-body pain level remained around NRS 0-2 for 4-5 days after each treatment session. Twelve weeks later, the NRS value of her whole-body pain and the staging improved to 3 and 1, respectively. She was able to resume housework. Although her depressed mood and anxiety remained, JFIQ decreased to 52 pt (Figure 2). Her medical condition stabilized, and we introduced her to an acupuncture clinic for maintenance therapy. Acupuncture was performed by a clinical physician with three years of acupuncture experience, and no adverse events occurred.

## Discussion

This case illustrates effective pain relief using *Hokushin-kai*, a traditional Japanese style of acupuncture and moxibustion [7], for a patient with a four-month history of FM. Previous research spanning five years and 1555 FM patients revealed that substantial improvement in pain was observed in about 10% and moderate improvement in about 15% of patients. FM severity worsened in 35% of patients, and pain worsened in 38% [10]. In the 2022 JFSA questionnaire survey [4] (n=339), only 1.5% of respondents stated they were able to work without problems, 15.9% were working just barely, 9.4% were unable to work, 25.4% were barely doing housework, and 18.6% found housework difficult. These data suggest that patients with FM are unlikely to go into spontaneous remission and that symptoms remain difficult to control. In addition, many FM patients experience difficulties in their daily lives. In our case, the whole-body pain level of the patient remained around NRS 0-2 after six weeks of acupuncture treatment. This seems to be a faster and greater reduction of pain than the previous randomized controlled trial (RCT) series [5,6]. We surmise some possible explanations.

In this presentation, we used an acupuncture treatment that was different from the TCM or Western techniques. A previous case report of a 35-year-old woman with a one-year history of FM who was treated with the *Hokushin-kai* style showed a quick decrease in pain (from NRS 10 to NRS 5) one month after acupuncture treatment began, and to NRS 0-3 after four months [11]. *Hokushin-kai* style acupuncture [7] is a traditional form of Japanese acupuncture and moxibustion. One characteristic is that only one or two needles were used for each treatment session and without manipulation or electricity. In the *Hokushin-kai* style, performing a detailed acupoint exam beforehand is very important before selecting the appropriate acupoints for treatment, and this strategy enables strong therapeutic effects with only one needle [7,12].

Furthermore, the recent concept of treatment-sensitive periods (termed ‘windows of opportunity’) for chronic pain prevention has been proposed, especially for central neuropathic pain [13]. This concept supports the importance of early treatment of FM. Conversely, no clinical study supports a delay in initiating acupuncture treatment for chronic FM pain, which is related to poor treatment prognosis, and many acupuncturists empirically know the limitations of acupuncture treatment. The mechanism of resistance to acupuncture treatment for chronic pain is still unknown. Interestingly, recent research indicating abnormalities in the peripheral nerves of the skin of FM patients has been conducted. Indeed, approximately 30-70% of FM patients have reduced intraepidermal nerve fiber density, and spontaneous activity in C-fibers is also seen [14,15]. Thus, this small-fiber pathology may explain one of the mechanisms of resistance to acupuncture treatment for chronic pain.

Another factor to consider is that our patient took an SSRI (paroxetine). Note that a Cochrane Database System Review concluded that there is no unbiased evidence supporting the use of an SSRI for treating pain, fatigue, and insomnia related to FM; however, they state it can be considered for treating depression in patients with FM [16]. Acupuncturists sometimes treat the same FM patients as medical doctors who prescribe SSRIs without each healthcare professional knowing what the other is doing in clinical practice, which is problematic. No clinical trials have evaluated the combined effects of SSRIs and acupuncture for FM, so such trials could be considered.

The Cochrane Database System Review concluded that amitriptyline, a tricyclic antidepressant (TCA), reportedly has a limited pain relief effect on FM [17]. In addition, an RCT for chronic FM patients (mean pain duration: 118.8 months) showed that the visual analog scale significantly improved from 8 to 5 after three months of acupuncture treatment compared with TCAs or exercise alone [6]. Therefore, combining antidepressants and acupuncture might reduce pain in FM patients.

Both acupuncture and SSRIs may improve pain by regulating neural activity in the serotonin system. Acupuncture was first used in ancient China. Its clinical effect is demonstrated by the insertion of a needle into specific parts of the body termed acupoints. A recent study revealed that the mechanism of pain relief enabled by acupuncture is associated with activating the descending pain pathway via increasing the level of spinal or brain 5-hydroxytryptamine (5-HT), norepinephrine (NE), and opioid peptides [18], although a few side effects such as malaise (in 7%) were reported [19]. Moreover, a clinical trial for FM patients showed that increasing serum serotonin values and improving pain relief were observed after treatment of acupuncture [20]. These data suggest that combining SSRI and acupuncture might have a synergistic effect on pain via the 5-HT pathway.

Further studies are required to establish whether the treatment prognosis is improved by combining SSRIs and acupuncture, treatment with a traditional Japanese acupuncture method, such as *Hokushin-kai*, or initiating acupuncture earlier for FM. A better understanding of the relationship between acupuncture treatment resistance and small fiber pathology is also needed.

## Conclusions

We described a case in which acupuncture using a traditional Japanese method (*Hokushin-kai*) greatly reduced the pain of FM patients receiving an early-phase intervention for their pain. Traditional Japanese acupuncture and moxibustion, such as the *Hokushin-kai* style, could be a viable treatment option for FM along with Western or TCM methods. Further research, such as a combination of SSRIs or early-phase intervention, is required to evaluate the better clinical effects of acupuncture for FM.
